# The Impact of Pitch and Timbre Cues on Auditory Grouping and Stream Segregation

**DOI:** 10.3389/fnins.2021.725093

**Published:** 2022-01-11

**Authors:** Yonghee Oh, Jillian C. Zuwala, Caitlin M. Salvagno, Grace A. Tilbrook

**Affiliations:** Department of Speech, Language, and Hearing Sciences, University of Florida, Gainesville, FL, United States

**Keywords:** auditory stream segregation, auditory grouping, pitch, timbre, fundamental frequency, spectral slope

## Abstract

In multi-talker listening environments, the culmination of different voice streams may lead to the distortion of each source’s individual message, causing deficits in comprehension. Voice characteristics, such as pitch and timbre, are major dimensions of auditory perception and play a vital role in grouping and segregating incoming sounds based on their acoustic properties. The current study investigated how pitch and timbre cues (determined by fundamental frequency, notated as F0, and spectral slope, respectively) can affect perceptual integration and segregation of complex-tone sequences within an auditory streaming paradigm. Twenty normal-hearing listeners participated in a traditional auditory streaming experiment using two alternating sequences of harmonic tone complexes A and B with manipulating F0 and spectral slope. Grouping ranges, the F0/spectral slope ranges over which auditory grouping occurs, were measured with various F0/spectral slope differences between tones A and B. Results demonstrated that the grouping ranges were maximized in the absence of the F0/spectral slope differences between tones A and B and decreased by 2 times as their differences increased to ±1-semitone F0 and ±1-dB/octave spectral slope. In other words, increased differences in either F0 or spectral slope allowed listeners to more easily distinguish between harmonic stimuli, and thus group them together less. These findings suggest that pitch/timbre difference cues play an important role in how we perceive harmonic sounds in an auditory stream, representing our ability to group or segregate human voices in a multi-talker listening environment.

## Introduction

Multiple sound sources are often simultaneously active in everyday listening environments. In a multi-talker listening environment, the auditory system must be able to distinguish the target voice from all the others and isolate it. This allows the listener to understand, process, and properly respond. This is an important ability that is not always easy, as sounds coming from multiple sources often blend together. This ability to focus one’s auditory attention on a single stimulus amidst several other competing stimuli is well known as the “cocktail party effect” (Cherry, 1953).

An auditory stream is the percept associated with grouping individual sounds together as a coherent whole by assigning its elements as incoming from the same source and belonging together (van Noorden, 1975; Bregman, 1978, 1990; Moore and Gockel, 2012). In the traditional auditory streaming paradigm using A-B pairings, in which A and B represent alternating tone bursts, the perception of a single-sourced stream from a sequence of sounds is termed fusion, or grouping. When perceived as more than one stream, this is termed fission, or stream segregation. The line of demarcation between these two processes is the fusion-fission boundary. Auditory grouping ranges, defined by this boundary, are useful in observing the amount of separation required for stream segregation to occur, and determining whether streams have their own distinct identity amongst interfering incoming sound sequences.

Many previous studies have shown that auditory grouping/stream segregation performance using A-B alternating complex tones can be influenced by major perceptual attributes, including pitch and timbre (van Noorden, 1975; Singh, 1987; Bregman et al., 1990; Hartmann and Johnson, 1991; Singh and Bregman, 1997; Vliegen and Oxenham, 1999; Cusack and Roberts, 2004). Pitch is a characteristic primarily determined by a sound’s frequency, representing how perceptually high or low a sound is based on a frequency scale (ANSI, 1994). In harmonic complex tones, pitch is the perceptual objective correlate of fundamental frequency (hereby referred to as F0). Vliegen and Oxenham (1999) found that listeners could generally segregate tones A and B into two perceptual streams when their F0s were about 4 semitones apart, whereas van Noorden (1975) found a smaller segregation boundary (around 1 semitone).

Another characteristic, timbre, is an interesting component of sound that is representative of sound quality. Timbre cannot be defined or measured by a single physical dimension. Rather, it can be thought of as the auditory element that distinguishes two sounds as being dissimilar even when they are equal in pitch, loudness, and duration (Bregman, 1990; ANSI, 1994). Although this is the widely accepted definition of timbre, it is still quite vague and leaves room for interpretation. Thus, the most appropriate way to measure a sound’s timbre will largely depend on the properties of the sound itself. Dynamic variations of the shape of either temporal or spectral envelopes have been known to have an important effect on the timbre (Grey, 1977). Hartmann and Johnson (1991) investigated the influence of two attributes of timbre on stream segregation. Their findings show that the temporal envelope shape makes no significant contribution to stream segregation, and spectral envelopes are more dominant factors than temporal envelope factors attributed to stream segregation. In contrast, Singh and Bregman’s (1997) results demonstrated that the timbre cue (specifically, the temporal envelope) can alter the extent of stream segregation. A more recent study (Cusack and Roberts, 2004) focused only on the temporal envelope for each harmonic component (termed spectral variation by the authors) and found enhanced segregation when the patterns of spectral variation differed between component A and B in a sequence.

A number of studies have explored the relative roles of both pitch and timbre cues in stream segregation performance. Both Singh (1987) and Bregman et al. (1990) focused on F0 and spectral envelope (spectral region in Singh, 1987; formant peak in Bregman et al., 1990) as representatives of the perceptual cues pitch and timbre, respectively. Their studies found that segregation abilities significantly increased as either F0 or spectral envelope differences increased and that F0 and spectral slope contribute independently to stream segregation. Our current study follows the same methodology by focusing on pitch and timbre in an auditory streaming paradigm. However, this study kept the physical parameter for pitch as F0 but changed the physical parameter of timbre to spectral slope, to represent the auditory streaming paradigm in another domain of pitch and timbre pairings.

**FIGURE 1:**
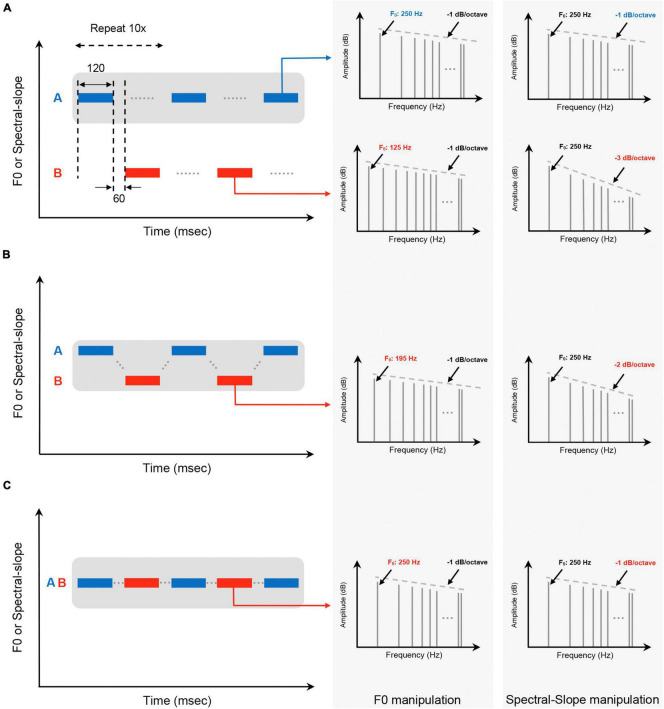
Schematic of stimuli used in the auditory grouping range measurement with example stimuli for **(A)** “two streams” perception, **(B)** “ringing” perception, and **(C)** “even” perception. Gray shaded areas in **(A–C)** indicate the boundaries of the grouping range around the stimulus A. The right two columns represent example harmonic stimuli of each percept in the F0 domain and the spectral slope domain. The F0 and spectral slope of each stimuli are also indicated.

Spectral slope depicts how rapidly the amplitudes of a sound’s successive parts decrease as their harmonic frequencies increase (see dashed lines in the right columns of Figure 1). In particular, spectral slope is representative of the sharpness of sound (Fastl and Zwicker, 2007); a steeper negative slope represents a duller sound (because it is generally less intense at higher frequencies), while a shallower negative slope represents a sharper sound (because it is generally more intense at higher frequencies). Luo et al.’s (2019) study discussed the changes in sound perception when the spectral envelope, as a measurement of timbre, is manipulated. Their study used words to relate these quality changes to perception, “dull-sharp, compact-scattered, colorful-colorless, full-empty.” Among these scales, *dull-sharp* carries the most variance and is an adequate relation to frequency limit and spectral slope (von Bismarck, 1974; Luo et al., 2019).

In the current study, we explore the perceptual cues of pitch and timbre while varying physical parameters F0 and spectral slope, respectively, in the harmonic complex stimuli to observe their effects on a normal hearing (NH) listener’s ability to group and segregate sound sources in an auditory streaming paradigm. We hypothesize that our results will show a similar trend as Singh (1987) and Bregman et al. (1990) found, in which greater differences in pitch and timbre cues will increase a NH listener’s ability to segregate two alternating harmonic sound sources. However, due to the multidimensional nature of timbre, and its dependence on other perceptual cues (pitch and loudness), we hypothesize that pitch and timbre cues will contribute interdependently to stream grouping and segregation performance. Additionally, we aim to further quantify the boundaries between grouping and segregation, defining the fusion-fission boundaries, in both F0 and spectral slope domains.

## Materials and Methods

### Subjects

This study was conducted in accordance with guidelines set forth by the Institutional Review Board (IRB) of the University of Florida to ensure the protection of human subjects. All employed methods were approved by that IRB. Twenty adult subjects (18 females; mean ages = 21.8 ± 1.2 years old) were recruited from the University of Florida. All subjects performed a hearing screening: (1) audiometric thresholds ≤ 25 dB hearing level (HL) from 125 to 8,000 Hz and (2) normal middle ear function as defined by 0.226-kHz tympanometry and air-bone gaps of 10 dB or less. All subjects scored ≥ 27 on the 10-min Mini Mental Status Examination (MMSE; Folstein et al., 1975), ruling out cognitive impairment that could potentially influence performance. All subjects who participated in this study passed both the cognitive screening and hearing screening.

### Stimuli and Procedures

All experiments were conducted in a single-walled audiometric sound booth. All stimuli were digitally generated at a sampling rate of 44.1 kHz with MATLAB (version R2018b, MathWorks, Natick, MA, United States), processed through an RME UFX + audio interface (RME Audio, Haimhausen, Germany), and presented via a frequency-equalized Yamaha HS5 loudspeaker (Yamaha, Shizuoka, Japan) which was positioned in the front hemifield, a distance of 1.5 m from the center of the listener’s head. The output of the loudspeaker was calibrated using a Brüel and Kjaer sound level meter with an A-weighting filter (Brüel and Kjaer Sound and Vibration Measurement A/S, Naerum, Denmark).

Auditory grouping ranges were measured with traditional ABAB auditory streaming sequences by manipulating two acoustic parameters of harmonic stimuli A and B: F0 and spectral slope. All the harmonics up to 4,000 Hz were included in a sine phase. Example harmonic structures of the stimuli used in this study are shown in Figure 1 (right two columns), indicating F0 as a function of frequency in hertz (Hz) and spectral slope as a function of frequency in octaves and amplitude in decibels (dB). We used the method of constant stimuli (Binder et al., 2009), in which stimulus A was fixed, while stimulus B was pseudo-randomly varied with multiple presentations (six repeats) in each trial. Stimuli A and B were repeated for a total of 10 presentations with a 120-ms stimulus and 60-ms interstimulus between presentations, organized into A-B pairings. All stimuli consisted of tones with 10-ms raised-cosine onset/offset ramps. The overall level of each stimulus was fixed at 65 dB sound pressure level (SPL).

Firstly, for the auditory grouping range measurement in the F0 domain referred to as the “F0 grouping range,” the F0 of stimulus A was fixed at 250 Hz. The F0 values of stimulus B were pseudo-randomly varied between 125 and 500 Hz, by a step size of 1/8 octave (resulting in an F0 of 125, 136, 148, 162, 176, 192, 210, 229, 250, 272, 297, 324, 353, 420, 458, or 500 Hz) within one block, so with 17 different stimuli, each block consisted of 102 sequences. Additionally, the spectral slope differences between stimuli A and B were manipulated in order to explore spectral slope dependent changes of the F0 grouping range; the spectral slope value of stimulus A was fixed at −1 dB/octave, while those of stimulus B was fixed at 0, −1, and −2 dB/octave. These ± 1-dB/octave spectral slope differences were the average spectral slope discrimination limens (DLs) for NH listeners, as reported from Luo et al.’s (2019) study. Secondly, for the auditory grouping range measurement in the spectral slope domain referred to as the “spectral slope grouping range,” the spectral slope of stimulus A was fixed at −1 dB/octave. The spectral slope values of stimulus B were pseudo-randomly varied between −3.1 and 1.1 dB/octave, by a step size of 0.3 dB/octave (resulting in an spectral slope of −3.1, −2.8, −2.5, −2.2, −1.9, −1.6, −1.3, −1, −0.7, −0.4, −0.1, 0.2, 0.5, 0.8, or 1.1 dB/octave) within one block, so with 15 different stimuli, each block consisted of 90 sequences. To explore F0-dependent changes of the spectral slope grouping ranges, the F0 differences between stimuli A and B were manipulated; the F0 value of stimulus A was fixed at 250 Hz, and that of stimulus B was fixed at 240, 250, and 270 Hz. These ± 1-semitone F0 differences (i.e., 240 and 270 Hz) were the average F0 DLs for NH listeners, which were also reported from Luo et al.’s (2019) study.

In summary, the F0 and spectral slope of stimulus A were fixed at 250 Hz and −1 dB/octave, respectively, throughout the whole experiment. The F0 grouping ranges between stimuli A and B were measured by varying F0s of stimulus B (125–500 Hz) and repeated at three different spectral slopes of stimulus B (0, −1, and −2 dB/octave). Similarly, the spectral slope grouping ranges between A and B were measured by varying spectral slopes of B (−3.1 to 1.1 dB/octave) and repeated at three different F0s of B (240, 250, and 270 Hz). A summary of the stimulus conditions was shown in Table 1. For reliability, each condition was tested twice and averaged to analyze grouping ranges in F0 and spectral slope domains.

**TABLE 1:** Experimental conditions of fundamental frequency (F0) and spectral slope of harmonic stimuli A and B.

Measurement	Parameter	A	B
F0 Grouping range	F0 (Hz)	250	Varied from 125 to 500
	Spectral slope (dB/octave)	−1	0
			−1
			−2
Spectral slope Grouping range	Spectral slope (dB/octave)	−1	Varied from −3.1 to 1.1
	F0 (Hz)	250	240
			250
			270

A three-alternative forced-choice task was used in this study. Subjects were instructed to choose one of three percepts after each F0/spectral slope pair presentation: “two streams,” “ringing,” or “even.” Figure 1 shows the schematic of the stimuli for each percept. To make these judgments, listeners were instructed to (1) listen to the tone sequences presented in each trial; (2) decide whether the tone sequences were perceptually grouped or segregated; (3) record their response by selecting the appropriate selection on the touch-screen monitor. It should be noted that, in this study, both “ringing” and “even” responses were categorized as a single (grouped) stream, because the tone sequences were perceptually close enough in pitch/timbre that the listener was unable to fully separate them from one another. If the listeners were able to fully separate the tone sequences, they were instructed to choose the “two streams” percept (segregation). Subjects were allowed to repeat stimulus presentations as many times as necessary to make a selection.

**FIGURE 2:**
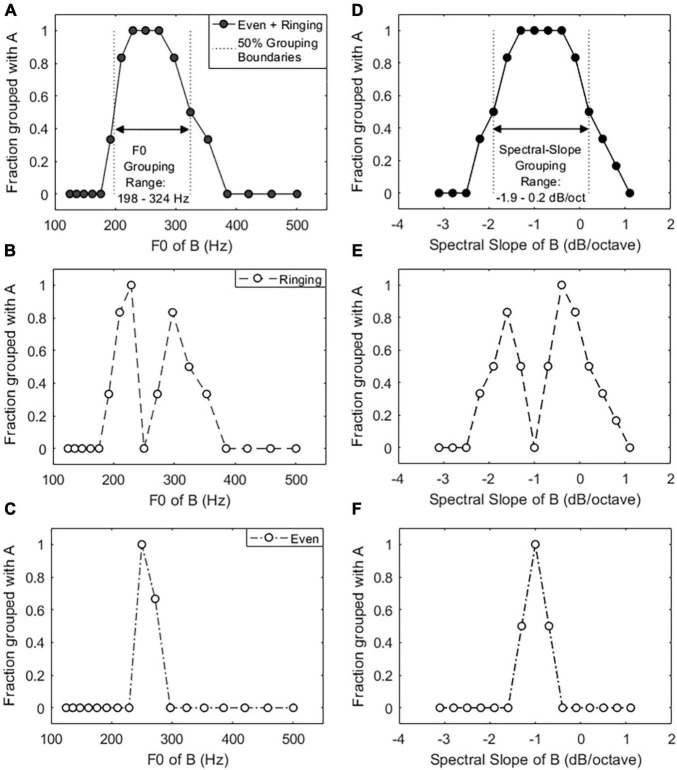
Example F0 grouping function **(A)** and spectral slope grouping function **(D)** calculated from the proportion of both the “ringing” responses **(B,E)** and “even” responses **(C,F)** (i.e., **A** = **B** + **C** and **D** = **E** + **F**). Vertical dotted lines in **(A,D)** indicate the 50% boundaries of the grouping range.

Averaged responses at each condition were analyzed to compute grouping functions. A “two streams” response has a value of 0 to indicate segregation, and “ringing” and “even” responses have a value of 1 to indicate grouping. Figure 2 shows a subject’s F0 grouping function (Figure 2A) and spectral slope grouping function (Figure 2D) including both “ringing” responses (Figures 2B,E) and “even” responses (Figures 2C,F). The “two streams” percept is associated with complete segregation between A and B tones, meaning that the listener does not perceive grouping of the two sound stimuli, as seen in the flat portions of Figures 2A–F located outside of the grouping ranges. A “ringing” percept is associated with grouping, leading listeners to be unable to completely separate the identities of the two tones into separate streams, expressed in Figures 2B,E with two peaks representative of complete grouping and complete segregation seen at 250-Hz F0 and −1 dB/octave spectral slope, respectively. An “even” response is indicative of stronger grouping, with perceptual components of the tones being indistinguishable from one another, creating a sharper grouping range (Figures 2C,F) with both tones A and B being presented at the same F0 (250 Hz) or spectral slope (−1 dB/octave).

Testing was usually conducted in 1–2 sessions totaling approximately 3 h, and ample breaks were provided to minimize test fatigue. Each subject was paid an hourly wage for their time. No prior experience with psychoacoustic research was required for participation, and a practice trial was provided to ensure familiarity with the procedure and the three possible percepts (two streams, ringing, and even). All statistical analyses were conducted in SPSS (version 25, IBM).

## Results

**FIGURE 3:**
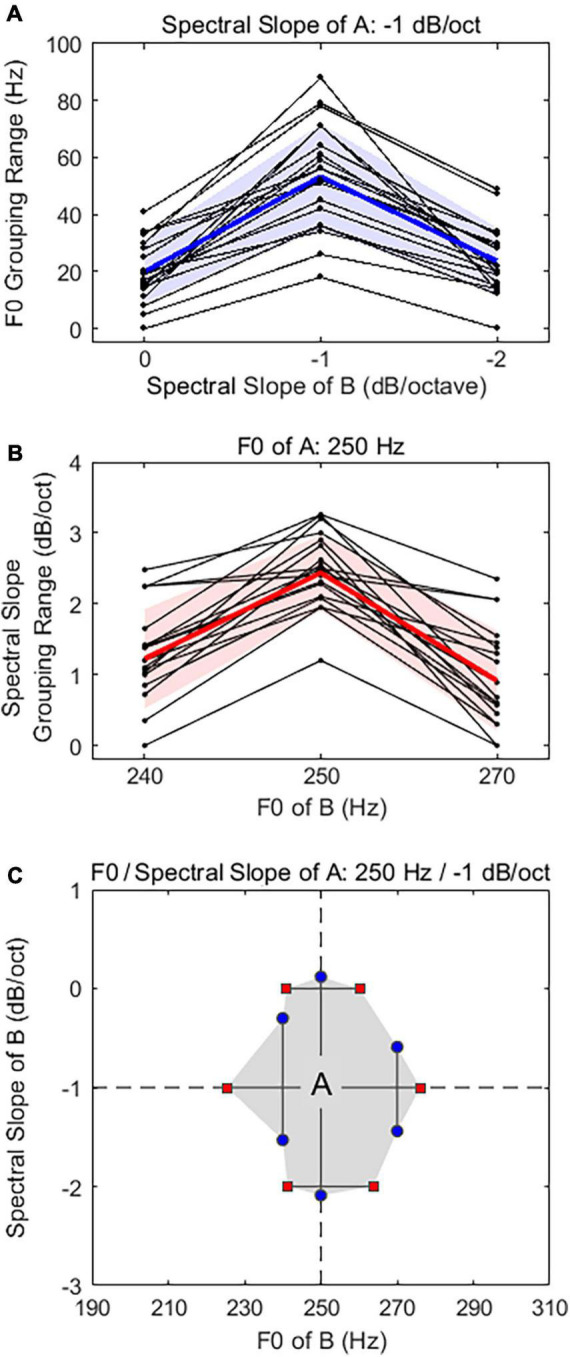
Individual and average F0 grouping range results **(A)** and spectral slope grouping range results **(B)**. In each panel, the solid thick line with the shaded area indicates the mean with standard deviation for all subjects, and thin solid lines show individual data. **(C)** Two-dimensional representation of auditory grouping ranges. The intersection of two dashed lines indicates the F0 and spectral slope of the stimulus A. Shaded region shows “grouping area” which are demarcated by upper and lower boundaries of the grouping ranges in F0 and spectral slope domains.

Figure 3 shows individual (thin lines) and averaged (thick lines) grouping range results in the F0 domain (Figure 3A) and in the spectral slope domain (Figure 3B). In the F0 domain, the F0 grouping range results were presented as a function of the spectral slopes of harmonic stimulus B. The results showed that the average F0 grouping range was 54 ± 18 Hz in the absence of spectral slope differences between A and B (i.e., −1 dB/octave spectral slope of B). Note that the spectral slope of A was fixed at −1 dB/octave. However, the F0 grouping ranges decreased as the difference in spectral slope of A and B increased (20 ± 10 Hz for the 0-dB/octave spectral slope of B; 24 ± 11 Hz for the −2-dB/octave spectral slope of B). A linear mixed model (LMM) analysis was used to analyze the data with the F0 grouping range as a dependent variable, the spectral slope of B as a fixed factor, and the subject as a random factor. The model specification was as follows: F0 Grouping Range ∼ 1 + Spectral Slope of B + (1 | Subject). The LMM results showed a main effect of spectral slope differences between harmonic stimuli A and B on F0 grouping ranges [*F*_(2, 38)_ = 85.8, *p* < 0.001]. *Post hoc* pairwise comparisons using Bonferroni correction showed that the F0 grouping ranges at the ± 1 dB/octave spectral slope differences between A and B were significantly lower than those in the absence of spectral slope differences (*p* < 0.001 for both cases), but no significant difference was observed between + 1- and −1 dB/octave spectral slope difference conditions (*p* = 0.645).

A similar trend was also observed in the spectral slope domain (Figure 3B). Note that the spectral slope grouping range results were presented as a function of the F0 of stimulus B. The results showed that average spectral slope grouping ranges were maximized at 2.3 ± 1.1 dB/octave in the absence of the F0 difference between A and B, however, the spectral slope grouping ranges decreased as the difference in F0 of A and B (250 Hz F0 in both A and B) increased (1.2 ± 0.9 dB/octave for the 240-Hz F0 of B; 0.9 ± 0.8 dB/octave for the 270-Hz F0 of B). A separate LMM analysis [model: Spectral Slope Grouping Range ∼1 + F0 of B + (1 | Subject)] showed a main effect of F0 differences between stimuli A and B on spectral slope grouping ranges [*F*_(2, 38)_ = 49.9, *p* < 0.001]. *Post hoc* pairwise comparisons using Bonferroni correction showed that the spectral slope grouping ranges at the ± 1-semitone F0 differences (i.e., 240 and 270 Hz) between A and B were significantly lower than those at the absence of F0 differences (*p* < 0.001 for both cases), but no significant difference was observed between + 1- and −1-semitone F0 differences conditions (i.e., 240 vs. 270 Hz: *p* = 0.169).

Figure 3C shows a two-dimensional representation (shaded area) of the average grouping range results with the lower and upper boundaries obtained in both F0 (horizontal lines) and spectral slope (vertical lines) domains. Here, each symbol indicates the upper and lower boundaries of the grouping ranges in F0 and spectral slope domains, estimated from the result grouping functions (see example grouping functions in Figures 2A,D). The intersection of two dashed lines indicates the stimulus A (250-Hz F0 and −1 dB/octave spectral slope). In the F0 domain (horizontal), the grouping boundaries were 225 and 280 Hz (−2.4 and 1.4 semitones relative to 250 Hz) in the absence of the spectral slope difference between stimuli A and B, 241 and 260 Hz (−0.9 and 0.5 semitones relative to 250 Hz) in the 0-dB/octave spectral slope of B, and 241 and 264 Hz (−0.9 and 0.8 semitones relative to 250 Hz) in the −2 dB/octave spectral slope of B. In the spectral slope domain (vertical), the grouping boundaries were −2.1 and 0.1 dB/octave in the absence of the F0 difference between stimuli A and B, −1.5 and −0.3 dB/octave in the 240-Hz F0 of B (−1 semitone relative to 250 Hz), and −1.4 and −0.6 dB/octave in the 270-Hz F0 of B (+1 semitone relative to 250 Hz). When the F0 and spectral slope values of the stimulus B are presented within the grouping area, a listener is likely to perceive a grouped A-B sequence (either ringing or even percepts). When they are presented outside the shaded area, a listener is more likely to perceive two segregated streams.

## Discussion

This study measured auditory grouping ranges in response to changes in F0 (pitch) and spectral slope (sharpness or timbre) of the harmonic stimuli. Results show that the average grouping ranges were maximized in the absence of the F0/spectral slope differences between complex tones A and B and decreased by 2 times as their differences increased to ± 1 semitone in the F0 domain and ± 1 dB/octave in the spectral slope domain. In other words, increased differences in either F0 or spectral slope allowed listeners to more easily distinguish between harmonic stimuli, and thus group them together less frequently. Generally, data follow trends similar to those of both Singh (1987) and Bregman et al. (1990) in which either a more similar F0 or a more similar spectral slope led to more instances of grouping of tone sequences as opposed to segregation.

However, the results in the current study demonstrated that tone grouping/segregation performance can be affected by F0 and spectral slope cues by varying the two interdependently. These findings are not in agreement with those of Singh (1987) and Bregman et al. (1990), who found that F0 and spectral shape can contribute independently to stream segregation. This discrepancy might be due to the manner in which perceptual features, especially timbre, are manipulated. The Singh (1987) study used complex tones consisting of four consecutive harmonics and varied the harmonic number of the lowest harmonic (i.e., spectral region) to manipulate timbre. The Bregman et al. (1990) study used thirteen harmonics and varied the location of a single spectral peak (formant) to manipulate timbre. The current study used complex tones consisting of harmonics up to 4,000 Hz (i.e., 8–32 harmonics depending on F0) and varied their spectral slopes to manipulate timbre.

Another difference is that the current study measured actual grouping functions with three clear perceptual choices (two streams, ringing, and even percepts). This allowed the boundaries to be quantified between grouping and segregation (fusion-fission boundaries) in both the F0 and spectral slope domains, furthering the previous study’s investigation into the normal auditory system. To our knowledge, this is the first study to demonstrate an important role of both pitch and timbre (sharpness) in auditory grouping and stream segregation performance in a normal auditory system with the actual quantitative fusion-fission boundaries in both the F0 and spectral slope domains. The results from previous studies showed the fusion-fission boundaries in the F0 domain were varied between 1 and 4 semitones (van Noorden, 1975; Vliegen and Oxenham, 1999), which is compatible with the current results (−2.4 to 1.4 semitones relative to 250 Hz) in the absence of spectral slope difference cues. However, the current results suggest that the fusion-fission boundaries in the F0 domain can be influenced (reduced) by spectral slope differences between harmonic stimuli A and B (−0.9 to 0.8 semitones relative to 250 Hz). Note that both previous studies measured segregation boundaries higher than the reference F0 of 100 Hz, and the current study measured them in both lower and higher than the reference F0 of 250 Hz. Further, similar trends were observed in the spectral-slope domain, which has not been reported in previous studies: the fusion-fission boundaries were maximized in the absence of F0 differences between harmonic components A and B (−2.1 to 0.1 dB/octave) and reduced with the F0 difference cues (−1.5 to 0.3 dB/octave).

The importance of pitch and timbre cues has been observed in previous studies (Smoorenburg, 1970; Allen and Oxenham, 2014; Luo et al., 2019), although none of them has explored this phenomenon within the auditory streaming paradigm. Smoorenburg (1970) revealed that spectral information was necessary for the detection of the low pitch within a complex tone, supporting the present idea that both pitch and spectral-related cues are important for the segregation/fission process to occur. Both Allen and Oxenham (2014) and Luo et al. (2019) found that pitch and sharpness perceptions are equally weighted in NH listeners by measuring their just-noticeable differences. Our findings suggest that the use of both pitch and sharpness simultaneously can enhance segregation performance in NH listeners. It can be inferred that human voices that are more similar in their fundamental makeup are more likely to be grouped together, represented in our study by minimal differences between harmonic stimuli A and B.

Studies that have utilized pure tone stimuli offer important insight into grouping/segregation patterns and overall speech perception within auditory streams (Mackersie et al., 2001; Mackersie, 2003; Hong and Turner, 2006). Their studies found that in NH listeners as well as listeners with hearing loss, broader grouping ranges of pure tones in an auditory stream were associated with poorer sentence perception. These findings were clear but came with the understanding that harmonic complexes, such as the human voice, required auditory cues that were not present in pure tones, including timbre. More streaming studies are needed in the realm of complex speech stimuli investigating the interdependency of pitch and timbre in order to better understand their implications, especially in those with hearing impairments.

Harmonic structures are generated by the vocal folds, and the overall harmonic shape is dependent on the shape of the vocal cavity and the articulators (Zhang, 2016). The current study changes F0 and spectral slope components, investigating grouping/segregation boundaries with stimuli more similar to speech rather than pure tone studies. Future investigation of the auditory streaming paradigm can be expanded to use the real vocal pitch and vocal timbre cues and test this phenomenon with hearing-impaired individuals and may help determine which mechanisms influence their personal grouping ranges. Hong and Turner (2006) tested NH listeners’ and CI users’ segregation abilities using pure tone auditory streaming. The trend seen for NH listeners was consistent with ours—increased frequency separation resulted in increased segregation. However, results varied more for CI users, with some performing similarly to NH listeners and others performing significantly poorer. According to the authors, this difference may be explained by the amount of nerve survival throughout the cochlea varying from person to person (Hong and Turner, 2006). Regardless of the reason for variance, understanding that some CI users struggle to segregate pure tones suggests further issues with segregating complex harmonic tones that are more representative of the human voice. Thus, we propose that further research regarding timbre should be conducted within this community, especially the interdependence between pitch and timbre cues that we investigated. Future research should include hearing impaired listeners in their subject pool to further investigate the interdependence of pitch and timbre in this population. This information could potentially increase the success of hearing assistive technology in multi-talker environments (i.e., cocktail party effect; Cherry, 1953).

## Conclusion

In this study, we explored the perceptual correlates of F0 and spectral slope (pitch and timbre, respectively) in the harmonic complex stimuli to observe a listener’s ability to group and segregate sound sources in an auditory streaming paradigm. Data collected from normal hearing listeners indicate that the manipulation of F0 and spectral slope differences produces a significant impact on reduced grouping frequency ranges, illustrating the potential influence of both voice pitch and timbre cues on auditory grouping/stream segregation performance. By finding the effect of these variables in a streaming paradigm we can apply this concept to the complex multi-talker listening environment experienced with the cocktail party problem. Further investigation with this effect for hearing impaired listeners could open the possibility for improved hearing assistive technologies to combat difficulty listening in these multi-talker listening environments.

## Data Availability Statement

The original contributions presented in the study are included in the article/supplementary material, further inquiries can be directed to the corresponding author/s.

## Ethics Statement

The studies involving human participants were reviewed and approved by the Institutional Review Board of the University of Florida. The patients/participants provided their written informed consent to participate in this study.

## Author Contributions

YO designed the experiments. CS, JZ, and GT performed the experiments. YO, CS, JZ, and GT analyzed the data, wrote the article, and discussed the results at all states. All authors contributed to the article and approved the submitted version.

## Conflict of Interest

The authors declare that the research was conducted in the absence of any commercial or financial relationships that could be construed as a potential conflict of interest.

## Publisher’s Note

All claims expressed in this article are solely those of the authors and do not necessarily represent those of their affiliated organizations, or those of the publisher, the editors and the reviewers. Any product that may be evaluated in this article, or claim that may be made by its manufacturer, is not guaranteed or endorsed by the publisher.
